# Partial rotational lattice order–disorder in stefin B crystals

**DOI:** 10.1107/S1399004714000091

**Published:** 2014-03-19

**Authors:** Miha Renko, Ajda Taler-Verčič, Marko Mihelič, Eva Žerovnik, Dušan Turk

**Affiliations:** a Department of Biochemistry and Molecular and Structural Biology, Josef Stefan Institute, Jamova 39, SI-1000 Ljubljana, Slovenia; b Centre of Excellence for Integrated Approaches in Chemistry and Biology of Proteins, Jamova 39, SI-1000 Ljubljana, Slovenia

**Keywords:** crystal disorder, rotational order–disorder, twinning, stefin B

## Abstract

At present, the determination of crystal structures from data that have been acquired from twinned crystals is routine; however, with the increasing number of crystal structures additional crystal lattice disorders are being discovered. Here, a previously undescribed partial rotational order–disorder that has been observed in crystals of stefin B is described. The diffraction images revealed normal diffraction patterns that result from a regular crystal lattice. The data could be processed in space groups *I*4 and *I*422, yet one crystal exhibited a notable rejection rate in the higher symmetry space group. An explanation for this behaviour was found once the crystal structures had been solved and refined and the electron-density maps had been inspected. The lattice of stefin B crystals is composed of five tetramer layers: four well ordered layers which are followed by an additional layer of alternatively placed tetramers. The presence of alternative positions was revealed by the inspection of electron-density score maps. The well ordered layers correspond to the crystal symmetry of space group *I*422. In addition, the positions of the molecules in the additional layer are related by twofold rotational axes which correspond to space group *I*422; however, these molecules lie on the twofold axis and can only be related in a statistical manner. When the occupancies of alternate positions and overlapping are equal, the crystal lattice indeed fulfills the criteria of space group *I*422; when these occupancies are not equal, the lattice only fulfills the criteria of space group *I*4.

## Abstract 2

Crystal lattice disorders are a phenomenon which may hamper the determination of macromolecular crystal structures. Using the case of the crystal structure of stefin B, identification of rotational order–disorder and structure determination are described.

## 1. Introduction   

In the last two decades, there has been an exponential growth in the number of protein crystal structures that have been deposited in the Protein Data Bank (PDB; Berman *et al.*, 2000 ▶). This increased deposition rate has been facilitated by advances in instrumentation, software development and novel high-throughput approaches that have been introduced in structural biology (Stevens *et al.*, 2001 ▶; Terwilliger, 2011 ▶). The increasing number of crystal structures has provided insight into many important biological processes which could not be provided by other approaches. With the increasing rate of structure determination, crystals with lattice disorders are more commonly observed; however, data are also discarded or lattice disorders remain undetected. The most common type of crystal lattice disorder is twinning (Yeates, 1997 ▶), although other less frequent crystal lattice disorders, as indicated by diffraction disorders, have also been observed (Helliwell, 2008 ▶). Merohedral twinning can be detected from scaling statistics and should now be routinely recognized and considered during refinement using software programs such as *SHELXL* (Sheldrick, 2008 ▶), *CNS* (Brünger *et al.*, 1998 ▶), *PHENIX* (Adams *et al.*, 2010 ▶) and *REFMAC* (Murshudov *et al.*, 2011 ▶). Despite wide awareness of twinning, there remain instances in which the twinning was not detected by the depositors. An analysis of the 11 367 structures with structure factors that were deposited before February 2004 indicated that there were at least 78 instances with a high degree of confidence of merohedral twinning (Lebedev *et al.*, 2006 ▶), whereas a recent analysis indicated that the number of twinned structures may be as high as 3% (Afonine *et al.*, 2010 ▶).

Among other crystal lattice disorders are order–disorder (OD) structures (also named crystals with lattice-translocation defects). In such crystals, successive crystal layers of molecules are shifted in alternative directions (Dornberger-Schiff, 1956 ▶; Dornberger-Schiff & Grell-Niemann, 1961 ▶). These OD structures can be detected during data collection because of the modulated intensities and linearly diffuse profiles of several classes of reflections. The OD phenomenon has also been observed in various protein crystals, and in a few instances has resulted in successful structure determination (Trame & McKay, 2001 ▶; Kamtekar *et al.*, 2004 ▶; Wang, Kamtekar *et al.*, 2005 ▶; Wang, Rho *et al.*, 2005 ▶; Hwang *et al.*, 2006 ▶; Rye *et al.*, 2007 ▶; Tanaka *et al.*, 2008 ▶; Zhu *et al.*, 2008 ▶; Hare *et al.*, 2009 ▶; Tsai *et al.*, 2009 ▶). Recently, Pletnev *et al.* (2009 ▶) reported the structure of the fluorescent protein FP480, in which the random distribution of alternatively orientated tetramers created a statistically averaged *I*422 symmetry. Pletnev and coworkers named this disorder a ‘rotational order–disorder structure’. In addition, the structure of human carbonic anhydrase was first identified as a rotational order–disorder structure (Robbins *et al.*, 2010*a* ▶); however, this identification was later corrected and refined as a structure with an alternative selection of molecules in the asymmetric unit (Robbins *et al.*, 2010*b* ▶).

During our attempts to crystallize various oligomeric forms of the cysteine protease inhibitor human stefin B, which is used as a model protein to study amyloid fibrillation (Zerovnik *et al.*, 2002 ▶) and amyloid-membrane perforation (Rabzelj *et al.*, 2008 ▶), several data sets were collected. The diffraction images indicated a nicely ordered crystal lattice. Yet, the data set from crystal 1 merged in space group *I*4, whereas the data set from crystal 2 was successfully merged in space group *I*422. In both instances, the first molecular-replacement solution resulted in an incomplete crystal lattice structure. Specifically, four well ordered layers were separated by an empty space that extended across the entire crystal. Here, we describe the structure solution, refinement and crystal packing of monomeric molecules of stefin B, which was identified as a crystal structure with rotational order–disorder packing.

## 2. Materials and methods   

### 2.1. Preparation and crystallization   

Stefin B was expressed as described previously (Rabzelj *et al.*, 2005 ▶). cDNA of stefin B coding for the complete sequence of the protein from Met1 to Phe98 was inserted into pET-11a vector. Stefin B was expressed in *Escherichia coli* BL21(DE3) cells after induction with IPTG and was purified using papain Sepharose and size-exclusion chromatography. The expressed protein differs from the wild-type protein (GenBank accession No. AAH10532.1) at one residue. Namely, the cysteine at position 3 was replaced by serine to prevent the formation of disulfide-linked dimers. Stefin B with this exact sequence has not been crystallized before.

The monomers were isolated using size-exclusion chromatography and concentrated to 19 mg ml^−1^ in 10 m*M* Tris–HCl, 100 m*M* NaCl pH 7.5.

The crystals, which were rectangular cuboids with dimensions of 0.2 × 0.2 × 0.5 mm, were obtained by sitting-drop vapour diffusion using 1 µl protein solution mixed with 1 µl reservoir solution (0.1 *M* CAPS buffer pH 10.5, 0.2 *M* Li_2_SO_4_, 2 *M* ammonium sulfate) and equilibrated against 1 ml of the same reservoir solution for a few days at 293 K. All crystals grew in identical conditions. The crystals appeared to be single and well shaped. Crystals were transferred into cryobuffer [reservoir solution supplemented with 20%(*v*/*v*) glycerol] for 5 s prior to flash-cooling in liquid nitrogen.

### 2.2. Data processing   

Two sets of diffraction data (data sets 1 and 2) were collected on beamlines BM14 at the ESRF, Grenoble and PX14.1 at BESSY, Berlin (Mueller *et al.*, 2012 ▶), respectively, at 100 K. The diffraction images were processed with *HKL*-2000 (Otwinowski & Minor, 1997 ▶) or *XDSApp* (Krug *et al.*, 2012 ▶; Kabsch, 2010 ▶).

**Figure 1:**
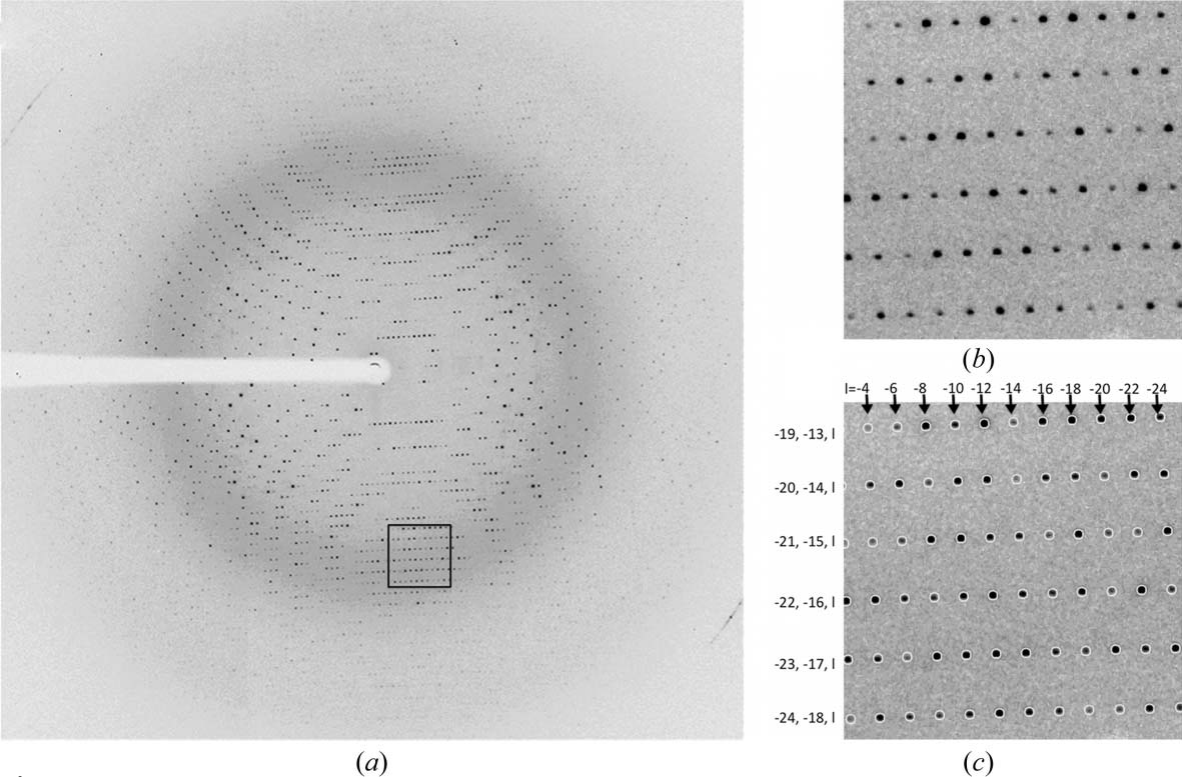
The diffraction pattern of stefin B crystal 1. (*a*) Complete diffraction image, (*b*) enlarged section and (*c*) the same enlarged section after integration with *HKL*-2000, containing the positions of predicted reflections with *h*, *k* and *l* indices shown.

Diffraction data were collected from several crystals; however, only two data sets were chosen for this structural analysis. Both crystals 1 and 2 diffracted to 1.8 Å resolution. 360 images with an oscillation of 1° per image were collected from crystal 1, while only 180 images (with the same oscillation) were collected from crystal 2 to 1.8 Å resolution owing to the diminishing diffraction quality. The observed diffraction patterns indicated the presence of a single crystal lattice in each data set with neither smeared nor satellite reflections present (Fig. 1 ▶, Supplementary Figs. S1 and S2). The data could be scaled in several space groups (*C*2, *F*222, *I*4 and *I*422), which all gave reasonable statistics, with *I*422 being the highest symmetry space group. The *R*_merge_ and numbers of rejections for both sets in various space groups are shown in Table 1 ▶. To compensate for the differences in the data completeness in higher resolution shells for the two crystals, only data truncated to 2.5 Å resolution were included in this comparison; elsewhere all data were used. The comparison shows that the data from crystal 2 could be scaled with approximately the same rejection rate in space groups *I*422 and *I*4, whereas the data from crystal 1 were merged in space group *I*422 with a considerably higher number of rejected reflections and an almost doubled *R*_merge_ compared with space group *I*4. The data-collection and scaling statistics for both sets are summarized in Table 2 ▶. Diffraction images are made available to the community by the TARDIS web server (http://tardis.edu.au/).

**Figure 2:**
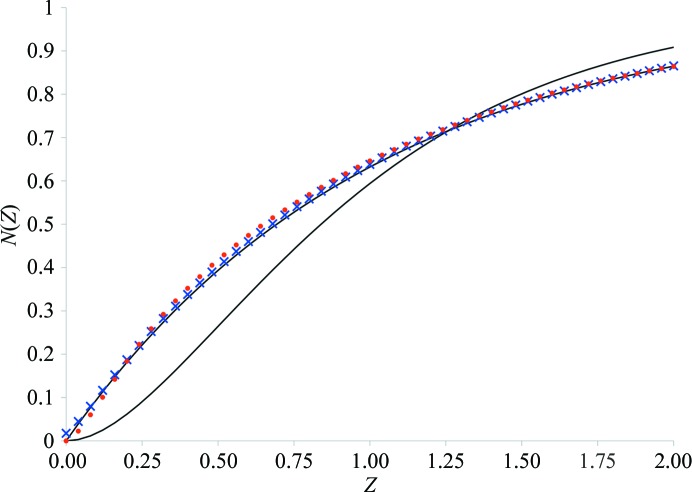
Twinning statistics. The experimental *N*(*Z*) plots calculated from the measured data for data set 1 (blue crosses) and data set 2 (red dots). The exponential and sigmoidal black lines correspond to nontwinned and perfectly twinned theoretical instances, respectively.

Because Table 1 ▶ shows intriguing scaling of the data, the data were checked for possible twinning. The intensity statistics showed a nearly perfect shape of the *N*(*Z*) plot (Fig. 2 ▶), a Wilson ratio 〈*I* ^2^〉/〈*I*〉^2^ of greater than 2 and 〈*L* ^2^〉 close to 0.5 for both sets (Table 3 ▶), excluding the possibility of merohedral twinning.

Twinning analyses with the *H*-test (Yeates, 1997 ▶) and the Britton test (Fisher & Sweet, 1980 ▶) did indicate perfect twinning. However, the use of these two tests is not suitable in this particular case. These two tests are based on the comparison of intensities within pairs of potentially twin-related reflections. Since the only possible twin law for space group *I*4 is *k*, *h*, −*l*, which corresponds to the symmetry operation present in space group *I*422, and since our data could also be merged in *I*422, these intensities were indeed almost equal. Therefore, resolution of the possible crystal twinning was postponed until analysis of the refined structure.

In addition, the largest off-origin peaks in the Patterson function are 5.4 and 5.8% of the origin peak for data sets 1 and 2, respectively, indicating the absence of significant pseudotranslation.

### 2.3. Molecular replacement in *I*422 using data set 2   

The structures were solved by molecular replacement with the *Phaser* crystallographic software (v.2.5.2; McCoy *et al.*, 2007 ▶) from the *CCP*4 suite (v.6.3.0; Winn *et al.*, 2011 ▶). Refinement was performed with *REFMAC*5 (Murshudov *et al.*, 2011 ▶) and *MAIN* (Turk, 2013 ▶). Additionally, real-space model corrections and molecular manipulations were performed with *Coot* (Emsley *et al.*, 2010 ▶) and *MAIN* (Turk, 2013 ▶). The data were verified with *phenix.xtriage* (Adams *et al.*, 2010 ▶).

Systematic absences clearly indicated the absence of a screw axis. Initial molecular replacement using the stefin B structure from the complex with papain (PDB entry 1stf; Stubbs *et al.*, 1990 ▶) with data set 2 in space group *I*422 found four positions in the asymmetric unit. Electron density for all four molecules was well defined. These four molecules formed four continuous layers that were parallel to the *ab* plane of the unit cell, which was intersected by one layer of empty space that was located directly on the twofold axis. Although a Matthews coefficient analysis did not allow us to ambiguously determine the number of molecules in the asymmetric unit (the Matthews coefficients were 3.31, 2.65 and 2.21 Å^3^ Da^−1^, respectively, for four, five and six molecules in the asymmetric unit), it was evident from the crystal packing alone that there should be more than four molecules in the asymmetric unit to build a three-dimensional crystal lattice.

The refinement of this partial model resulted in an *R* factor of 0.30 with *B* factors of between 20 and 30 Å^2^ for all four molecules. Visual inspection of electron-density maps of the molecular-replacement solution with four stefin B molecules revealed uninterpreted density within the empty layer. However, the resulting density maps were not clear enough to build the missing molecules. In part, this was a consequence of the potential overlap that resulted from the twofold crystal symmetry axis that passed through this empty layer. Therefore, we decided to solve the structure by molecular replacement in space group *P*1.

### 2.4. Molecular replacement in *P*1 using data set 1   

Molecular replacement with a monomer as a search model resulted in 32 positions, which formed eight tetrameric rings in four continuous layers and an empty layer. Additional molecular-replacement attempts using the positions of the first eight tetramers as a fixed solution and a tetramer as a search model resulted in placement of the ninth and tenth tetramers. Refinement of this model resulted in an averaged *B* factor of between 25 and 30 Å^2^ for the eight initially placed tetramers, whereas for the ninth and tenth tetramers the *B* factor was significantly higher (>50 Å^2^).

**Figure 3:**
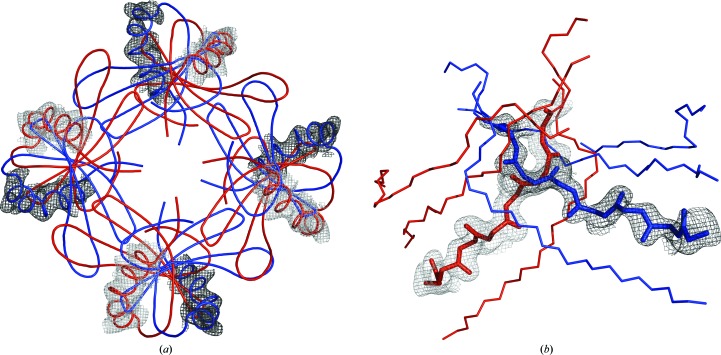
Density evidence for structural disorder. The pairs of overlapping molecules 0*A*, 0*B* and 5*A*, 5*B* are shown in red and blue, respectively. Score OMIT maps around molecules *A* and *B* are shown in grey and black, respectively. (*a*) Score OMIT 2*F*_o_ − *F*_c_ map around the helices of tetramers 0*A* and 0*B*. The score OMIT map was calculated by omitting both overlapping tetramers 0*A* and 0*B*. (*b*) Score OMIT *F*_o_ − *F*_c_ map for intersecting strands of molecule 0. The OMIT maps were calculated by omitting the 0*A* and 0*B* tetramers/molecules separately and were combined into one image.

Owing to the weak density in the region of the ninth and tenth tetramers, we inspected the position of the additional tetramers using score maps as implemented in *MAIN* (Turk, 2013 ▶). These maps confirmed that the placement of the ninth and tenth tetramers was indeed correct; however, the score maps, as well as the density around the models (shown in blue in Fig. 3 ▶ *a*), revealed some additional unexplained density which indicated the presence of additional helices. Because the stefin B structure contains only one helix, these additional unassigned helical density regions could only be explained by two additional tetramers which overlapped with the already positioned ninth and tenth tetramers by twofold symmetry.

Because the coordinate-system origin of space group *P*1 was chosen to superimpose with the solutions for the origins of space groups *I*4 and *I*422, we could exploit the twofold rotational axis which was present in the empty layer in the *I*422 space group, and modelled the alternate conformations of the ninth and tenth monomers. Indeed, in these alternative positions the newly placed helices of the models that are shown in red perfectly superimposed on the unassigned helical density (Fig. 3 ▶ *a*).

### 2.5. Refinement and model rebuilding   

Occupancy refinement with *REFMAC* against data set 1 was performed by screening different occupancies of the alternatively positioned molecules and by comparing their average *B* factors after refinement until a match was found. The occupancy refinement implemented in *MAIN* was performed by defining the two overlapping molecules as members of one overlapping group and by restraining the average *B* factor of these two molecules to the rest of the structure.

Structure solution in space group *P*1 revealed that the entire structure, which included the alternatively placed tetramers, still corresponds to the symmetry operators of space group *I*4. Therefore, to continue, refinement was performed in steps in space group *I*4 using NCS restraints between individual stefin B molecules. Firstly, only eight molecules (1–4 and 6–9) in the four initial layers were refined, and solvent molecules in their vicinity were added. The stefin B molecules in layer 0 (molecules 0*A*, 0*B*, 5*A* and 5*B*) were then added in both overlapping positions, with approximate occupancies deduced from their correlation with the electron-density maps using *MAIN*. The structures of the alternatively placed molecules were not manually corrected owing to ambiguous density; however, these structures were updated from the other molecules using the NCS operators. After refinement and the further addition of solvent molecules, occupancy refinement was performed by two independent approaches as described in §[Sec sec2]2. Both approaches gave similar results. The corresponding occupancies are summarized in Table 4 ▶. After a few additional cycles of manual structure improvement and refinement, the final *R*_work_ and *R*_free_ factors were 0.19 and 0.23, respectively (Table 5 ▶).

The electron densities for the alternatively placed molecules 1 and 6 were well defined in the β-strand and helix regions (as confirmed by the OMIT maps in Fig. 3 ▶ *b*), whereas the density in the loop regions was weaker. Additionally, there are some peaks that overlap with both alternatively placed molecules, which are most likely to correspond to partially occupied solvent molecules (not modelled).

The coordinates and structure factors for data set 1 were deposited in the Protein Data Bank under accession code 4n6v, whereas a download link for diffraction images and the partially refined structure of data set 2 are included in the Supporting Information.

## 3. Results   

### 3.1. Structure description   

The crystals of human stefin B contained the complete sequence of the protein from Met1 to Phe98. The *P*1 unit cell contains 40 molecules, whereas the *I*4 and *I*422 cells contain 80 molecules, with ten or five molecules in the asymmetric unit, respectively. The stefin B molecules share the cystatin fold, which is similar to that determined in the crystal structure of the complex with papain and which is composed of a five-stranded β-sheet that is packed against an α-helix. Cystatins are wedge molecules that utilize the N-terminus and two binding loops for binding into the active-site cleft of cysteine proteases (Stubbs *et al.*, 1990 ▶).

The non-overlapping stefin B molecules are well defined by the electron-density maps. The density did not enable us to ambiguously model the first seven residues that were positioned in the proximity of the fourfold symmetry axis. The Pro36 residue and Pro74–Pro79 loop are well defined in molecules 4 and 6 only, whereas in the other molecules their positions and conformations are not unambiguous; therefore, these characteristics were acquired from molecule 6 using the NCS operators. A total of 19 residues were modelled in alternate conformations. Residues Val48 and Asp77 are Ramachandran plot outliers. Val48 is unambiguously defined by the electron-density maps, whereas Asp77 lies in an less ordered region between Pro74 and Lys78 which has continuous density corresponding to the main chain. However, the density maps are rather featureless and do not enable exact positioning of the C^β^ and O atoms. Interestingly, an energetically unfavourable conformation of Val48 lies in the region where domain swapping occurs (Jenko Kokalj *et al.*, 2007 ▶).

All eight non-overlapping molecules are highly similar. The r.m.s.d. of all C^α^ atoms between any pair of these structures is lower than 0.32 A. When compared with the structure of stefin B in complex with papain (Stubbs *et al.*, 1990 ▶), the r.m.s.d.s are slightly higher and are in the range between 0.44 and 0.52 A.

The structure that was determined from crystal 2 is identical to that of crystal 1; however, the occupancy of the alternatively placed molecules is different (close to 0.5 for all four molecules), which allows scaling in space group *I*422 with a low number of rejections. The structure from crystal set 2 was not deposited owing to the lower quality of the diffraction data, which resulted in a less well defined structure. In particular, the areas of the first and second binding loop around residues Pro36 and Pro79 were not visible in the electron-density maps.

### 3.2. Crystal packing   

The crystal lattice is composed of monomers that are packed in tetrameric rings (as also called tetramers) positioned on top of each other along the fourfold crystallographic symmetry axis. When viewed from the side, the tetramers have a plate-like shape; therefore, we marked these tetramers with the symbol ‘(’.

In tetramers, the first binding loop, 46-QVVAG-50, packs against the groove that was formed at the side of the next molecule in the ring. The disordered N-termini fill the area in the centre around the fourfold crystal symmetry axis, whereas the residues of the second binding loop (74-PHENKP-79) are positioned on the external surface of the tetramer. The first binding loop is the area where the domain swap was observed in the domain-swapped dimeric structure of human cystatin C (Janowski *et al.*, 2001 ▶) and in the structure of the domain-swapped tetramer of human stefin B (Jenko Kokalj *et al.*, 2007 ▶.) Additionally, the second binding loop in stefin B was involved in the tetrameric loop exchange by the handshake mechanism of His75 in the latter structure. Hence, the packing that is described here does not contain any similarity to these packing and swapping mechanisms. Additionally, assembly analysis using *PISA* (Krissinel & Henrick, 2007 ▶) indicates that the tetramers are not a biologically relevant assembly.

**Figure 4:**
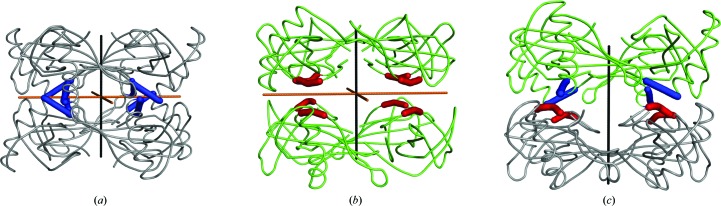
Three types of octamers are shown. (*a*) Octamer ‘)(’, (*b*) octamer ‘()’ and (*c*) octamer ‘((’. Green and black lines represent twofold and fourfold rotational axes, respectively. Molecules from octamers in (*a*) and (*b*) are shown in grey and green, respectively. The octamer in (*c*) is at the interface of the the octamers in (*a*) and (*b*). The colours of the molecules correspond to the colours in the octamers in (*a*) and (*b*). The fourfold rotational axis is crystallographic, whereas the twofold axes are point symmetry operations relating to the molecules in the octamers in (*a*) and (*b*), but not that in (*c*). The two regions that are involved in the interactions between the tetramers are shown in blue (11-PATA-14) and red (85-YQTN-88).

Pairs of tetrameric rings are packed together forming three types of octamers, two of which have 422 point symmetry. The first 422-type octamer (grey, Fig. 4 ▶ *a*) is formed from two pairs of tetrameric rings of molecules 1–2 and 8–9 [denoted ‘)(’], whereas the second 422-type octamer (green, Fig. 4 ▶ *b*) is formed from two pairs of tetrameric rings of chains 3–4 and 6–7 [denoted ‘()’]. Both twofold noncrystallographic symmetry axes of the ‘()’ and ‘)(’ octamers lie parallel to the *ab* plane. In the first type of octamer the twofold axis contacts are in the region 11–14 with the sequence PATA, whereas in the second type the twofold axis contacts are in the region 85–88 with the sequence YQTN. These two interacting regions are positioned on opposite sides of the stefin B molecules and are marked in red and blue, respectively, in Fig. 4 ▶. Hence, the stacking of the two tetrameric rings differs in these two types of octamers.

At the interface between the 422-type octamers, layers from two neighbouring tetramers build the third octamer type, in which the PATA and YQTN regions interact with each other [denoted ‘((’; Fig. 4 ▶ *c*]. Together, these octamers form stacks, which are termed stacks 1 and 2. The cross 422-type octamer interaction, PATA–YQTN, is characterized by a hydrogen bond between the NH group of Ala14 and the carbonyl group of Tyr85, whereas no hydrogen-bonding contacts are present in the PATA–PATA and YQTN–YQTN interfaces. Horizontally, the two antiparallel stacks of octamers make contacts in the outer tetramer layers 1 and 4, whereas the molecules from the intermediate two tetrameric layers 2 and 3 interact only vertically with molecules within the stack.

**Figure 5:**
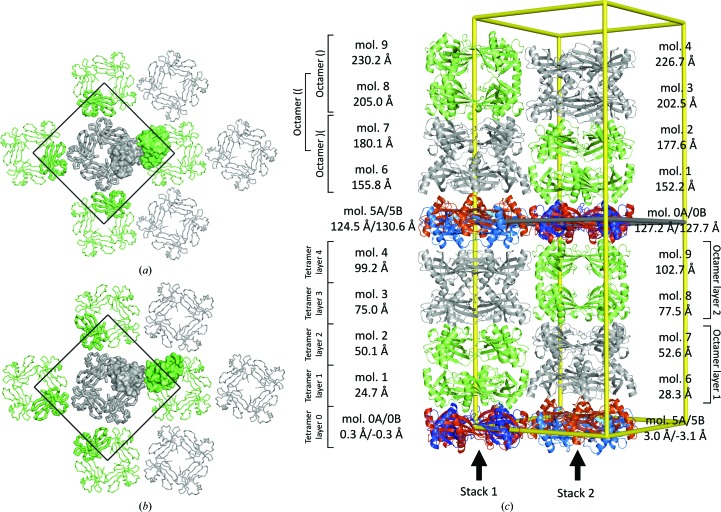
Crystal lattice organization. (*a*, *b*) Tetramer layers 1 and 2 of the crystal expanded into the surroundings by crystal symmetry operators. Molecules 1 and 2 are shown in green and molecules 6 and 7 are shown in grey, which correspond to the colours that are used in Fig. 4 ▶. The unit cell along the frame of *a* and *b* is shown with black lines. (*c*) Packing of the stefin B molecules in the *I*4 unit cell showing molecules, layers and octamers. Stefin B molecules in octamers ‘()’ and ‘)(’ are shown in green and grey, respectively. Alternatively placed tetramers 0*A*, 0*B*, 5*A* and 5*B* are shown in blue, red, light blue and light red, respectively. Molecules and tetramer layers are marked with numbers, octamers with braces and stacks with arrows. The average coordinate along the *z* molecule is shown for each molecule. The green line represents the position of the twofold rotational axis, which corresponds to the crystallographic twofold axis in space group *I*422 and relates to the alternatively placed pairs of tetramers 0*A*–0*B* and 5*A*–5*B*.

In the crystal (Fig. 5 ▶), the 422-type octamers build two octamer layers which correspond to the four vertical layers of tetramers that were initially found by molecular replacement. These two octameric layers are packed together such that two pairs of octamers pack antiparallel within each layer, with each vertical pair in a different stack. In these alternative positioning of octamers, the green octamers always neighbour grey octamers and *vice versa*. The same octamer type always has five octamer neighbours of the other type, specifically four around it within the same layer and one above or below within the same stack. The octamers in the layers are not related by proper NCS symmetry. A view perpendicular to the *ab* plane of the crystal into tetramer layers 1 and 2 shows (Figs. 5 ▶ *a* and 5 ▶ *b*) that the tetramers are oriented differently. However, in the crystal, the two octamer layers are related by the symmetry operation, which corresponds to the twofold axis that is present in space group *I*422.

As shown in Fig. 5 ▶(*c*), the average positions of the C^α^ atoms of two tetramers in the stack are 25.0 ± 0.5 Å apart; however, the centres of the molecules in the stacks are shifted 3 Å in opposite directions along the *c* axis, which corresponds to the *z* coordinate. The centre of molecule 9, which is the last in the second stack, is at 103 Å, which is ½*c* − 25 Å, whereas the centre of molecule 4, which is the last in the first stack, is at 100 Å; thus, the centre of molecule 4 is 3 Å lower than ½*c* − 25 Å. The same difference is found between the *z* coordinates of the centres of molecules 1 and 6. However, in the *ab* plane at *z* = 0 and *z* = *c*/2 the two stacks provide different spacing, which is reflected in the *z* coordinate of the molecules in the overlapping layer. In layer 0, there is less space between the first stack than between the second stack. As a consequence, the centres of overlapping molecules of the first stack, 0*A* and 0*B*, are at 0.1 and −0.1 Å, respectively, whereas the centres of overlapping molecules of the second stack, 5*A* and 5*B*, are 2.8 and −2.8 Å apart, respectively. At *z* = *c*/2, the situation is the reverse.

**Figure 6:**
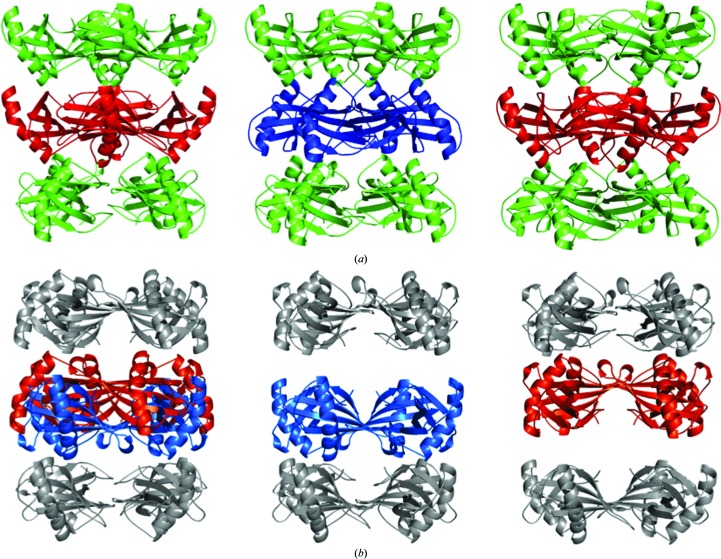
Observed orientations of the alternately positioned tetramers 0 (*a*) and 5 (*b*). The overlaid configuration is shown on the left, whereas the packing of individual molecules is shown in the centre and on the right. The colour code from Fig. 5 ▶ is used. For the nomenclature of the tetramers, see Fig. 5 ▶.

In layers 0 and 5, a direct vertical connection between two stacks is formed by tetramer 0 (Fig. 6 ▶ *a*), which makes a connection between tetramers 9 and 1. Owing to the crystal symmetry between the connecting molecules 1 and 9, a symmetrical interaction of the PATA regions from both of the tetramers is available. Therefore, tetramer 0 has two possible energetically equivalent positions, making a ‘)(’ octamer with tetramer 1 and a ‘((’ octamer with tetramer 9 (Fig. 6 ▶ *a*, centre) or *vice versa* (Fig. 6 ▶ *a*, right). In contrast, tetramer 5 does not connect both stacks owing to the 6 Å separation of the stacks (Fig. 6 ▶ *b*).

Therefore, tetramer 5 has two possible equivalent positions, assembling a ‘((’ octamer with tetramer 4 with no contacts with tetramer 6 (Fig. 6 ▶ *b*, centre) or *vice versa* (Fig. 6 ▶ *b*, right).

Although tetramer 0 directly connects both stacks vertically, the increased separation between tetramers 4 and 6 allows tetramer 5 to choose between the matching ‘()’ or cross ‘((’ interfaces. The crystal packing suggests that the hydrogen bond that bridges the ‘((’ PATA–YQTN packing is the favoured attachment; however, the ‘((’ interface cannot be excluded. The higher average *B* factor of tetramer 5 and the lower quality of the corresponding electron-density map around these molecules suggest that they are the consequence of the lack of the second crystal contact. As the crystal lattice stability does not depend on these molecules, one can not exclude the possibility that site 5 is not fully occupied or that there are four possibilities for the packing of tetramer 5 into this region.

Hence, the analysis suggests that packing alone is not responsible for differentiation between the two possible space groups. When the occupancy of the overlapping tetramers 0*A*, 0*B* and 5*A*, 5*B* is equal, then the twofold rotational axis (shown with a green line in Fig. 5 ▶ *c*) becomes the crystallo­graphic twofold axis, thereby changing the space group from *I*4 to *I*422, corresponding to crystal 2.

## 4. Discussion   

The determination of macromolecular structures from crystals that exhibit crystal lattice irregularities other than twinning requires additional attention because there is currently no standardized protocol and only a few instances have been reported. The match between the predicted and observed diffraction spots of the stefin B crystals during data processing in space group *I*422 indicated that there is only a single, well ordered crystal lattice. In addition, a superlattice found no support in the diffraction patterns (Fig. 1 ▶, Supplementary Figs. S1 and S2). Initial molecular-replacement attempts in space groups with higher symmetry (*I*422, *I*4, *F*222 and *C*2) resulted in unreasonable crystal packing; therefore, the most important parts of the structure determination were performed in space group *P*1. This refinement confirmed that the space group should be at least *I*4.

Nevertheless, potential merohedral twinning in space group *I*4 which used the twofold symmetry operator (*k*, *h*, −*l*) had to be explored.

Merohedral twinning was initially discarded by the twinning tests, which were based on the intensity statistics as shown in Fig. 2 ▶ and Table 3 ▶. Because the positions of eight of the ten molecules in the asymmetric unit of space group *I*4 were consistent with the *I*422 symmetry, the tests may not be sensitive enough to indicate twinning; therefore, we decided to analyze the crystal packing to find support for the merohedral twining or to reject this phenomenon.

### 4.1. Perfect twinning or partial rotational order–disorder  

**Figure 7:**
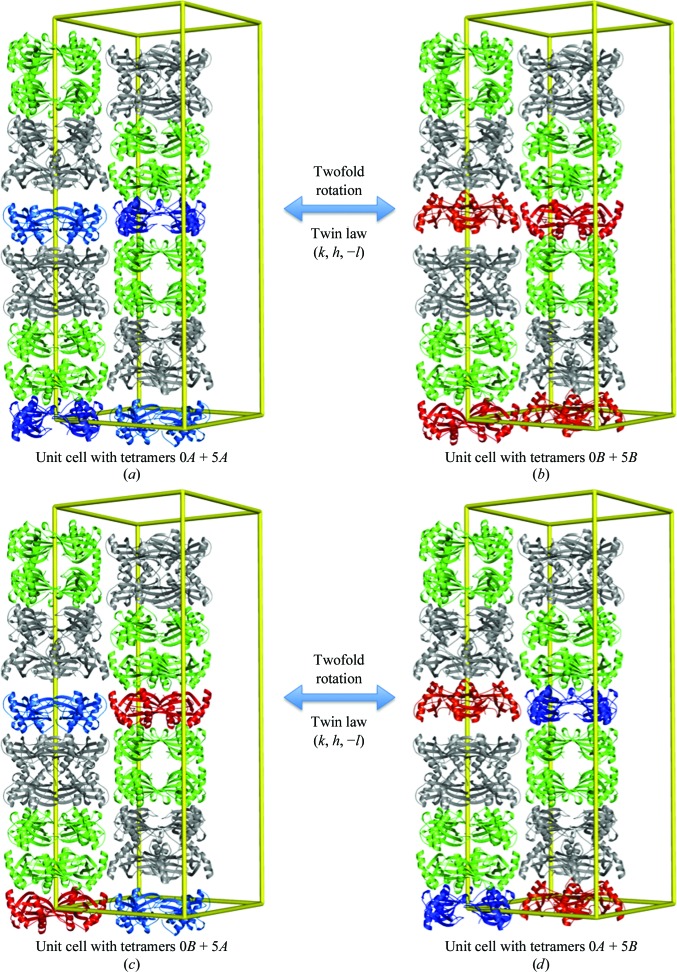
Two possible twinning scenarios. The colour of the molecules corresponds to the colour code used in Fig. 5 ▶.

Twins are regular aggregates consisting of crystals of the same species joined together in some definite mutual orientation

(Giacovazzo, 2002 ▶). Based on this definition, we could produce two possible twinning scenarios in space group *I*4 using the twofold symmetry operator (*k*, *h*, −*l*). Both twinning scenarios do not interfere with the four well defined layers of tetramers, as they are related by the twofold rotation axis, which is crystallo­graphic in the case of the potential space group *I*422. Only the positions of tetramers in layers 0 and 5 are affected by it. In the first scenario, the tetramers 0*A* and 5*A* shown in light and dark blue in Fig. 7 ▶(*a*) are in the twinned domain transformed by twofold rotation to the tetramers 0*B* and 5*B* shown in orange and red in Fig. 7 ▶(*b*). In the second scenario, the tetramers 0*B* and 5*A* shown in red and light blue in Fig. 7 ▶(*c*) are transformed to the tetramers 0*A* and 5*B* shown in dark blue and orange in Fig. 7 ▶(*d*). When the structures from the first or second scenario are merged, the final result is the same averaged crystal structure in both instances: the unit cell contains a pair of four well defined layers and two partially occupied layers composed of two pairs of tetramers 0*A* and 0*B*, 5*A* and 5*B*.

In the first twinning scenario, the occupancy of each pair of molecules 0*A*–5*A*, which are shown in blue in Fig. 7 ▶, and of molecules 0*B*–5*B*, which are shown in red, is identical, whereas in the second scenario the pairs with the same occupancy should be 0*B*–5*A* and 0*A*–5*B*. The occupancies of these molecules refined against the data for crystal 2 are 0.53, 0.47, 0.51 and 0.49 for molecules 0*A*, 0*B*, 5*A* and 5*B*, respectively. One might disregard the small differences, consider the occupancies to be equal and conclude that merohedral twinning is a plausible explanation for the crystal structure from data set 2 (which could be merged in space group *I*422 without considerable rejection of data). However, one cannot disregard the differences between the occupancies of the same molecules in crystal 1, which were 0.58, 0.42, 0.51 and 0.49 for molecules 0*A*, 0*B*, 5*A* and 5*B*, respectively. These differences are too large to accept the potential twinning scenario. Hence, we can conclude that the observed crystal disorder is not twinning but rotational order–disorder which was produced by random combinations of unit cells with different positions of tetramers 0 and 5 (as shown in Fig. 7 ▶) in an otherwise well arranged crystal network.

### 4.2. How can such disorders be identified?   

After a sufficient number of instances have been elaborated, the protocol may then be standardized; however, our report is only the second of such a crystal lattice disorder. The main question is how soon in the crystal structure determination can such a crystal disorder be detected and be properly considered: at the analysis of diffraction data, when solving the phase problem or during model building and refinement?

In the work of Pletnev *et al.* (2009 ▶), the presence of extremely weak and diffuse reflections and an impossibly small asymmetric unit indicated the presence of a type of crystal disorder, whereas in our instance the diffraction images did not provide any indication of the presence of an additional crystal lattice nor was crystal disorder evident from the data processing and its analysis. Data sets from both stefin B crystals could be scaled in several space groups with acceptable scaling statistics.

Molecular replacement already worked satisfactorily in space group *I*422. Only after inspection of the crystal packing did it become apparent that the solution resulted in packing which could not produce a stable three-dimensional connected crystal lattice. Returning to the *P*1 space group enabled us to build a stable crystal lattice; however, the refinement resulted in significantly higher *B* values and significantly more ambiguous densities of the two last placed tetramers. Only the inspection of score maps, as implemented in *MAIN*, enabled us to determine the alternative positions of the last two tetramers. Hence, visual map inspection, together with the score maps, which can average local density by the convolution theorem, were crucial steps in identifying the double occupancy within a layer of the crystal lattice.

The final conclusion regarding the correct space group and potential crystal twinning was deduced from comparison and analysis of the occupancies of overlapping molecules. This result indicates that the structures had to be refined in alternative space groups and that the structural data had to be analyzed before a final conclusion could be made regarding the correct space group and twin operators. To conclude, the analysis of each step, and all of them together, was crucial for determining this crystal structure with crystal lattice order–disorder.

## 5. Conclusions   

The crystal structure of stefin B in space group *I*4 demonstrates that protein crystals can contain a partial rotational order–disorder structure. In such instances, only part of the molecules in an asymmetric unit is present in several overlapping orientations. Such an ordered disorder cannot be detected in the diffraction pattern or by scaling statistics owing to the regular crystal network of the rest of the structure.

In macromolecular crystallography, it is a common practice to discard crystals that do not produce satisfactory solutions (such as an empty layer) and to continue with data collection from other crystals or even return to the wet laboratory until a crystal is found that enables the smooth resolution of a crystal structure. Perhaps this work will encourage crystallo­graphers to realise that different disorders may be more frequent than previously thought and that the strategy to continue with the data collection from multiple crystals with different percentages of disorders until a crystal with a low percentage of irregularities is found is not the only route to structure solution.

## Supplementary Material

PDB reference: stefin B, 4n6v

Supporting Information.. DOI: 10.1107/S1399004714000091/yt5064sup1.pdf

## Figures and Tables

**Table 1:** *R*_merge_, number of rejected reflections, total number of reflections and percentage of rejected reflections for sets 1 and 2 in various space groups A default error scale factor of 1.3 and an error zone of 0.03 were used in scaling with the *HKL*-2000 suite. To compensate for the differences in the data completeness in higher resolution shells between the two crystals, only low-resolution data truncated to 2.5 Å (20–2.5 Å) were used in this analysis. The diffraction data and scaling statistics tables for the data from crystals 1 and 2, truncated at 2.5 Å, in all of the mentioned space groups are available as Supplementary Tables S1 and S2.

	Data set 1				Data set 2			
	*R*_merge_ (%)	Rejections	Total	% rejected	*R*_merge_ (%)	Rejections	Total	% rejected
*I*422	7.2	24050	598277	4.02	6.6	3136	309292	1.01
*I*4	4.7	490	621836	0.08	6.2	2750	309679	0.89
*F*222	7.0	24919	590759	4.22	6.1	2272	310502	0.73
*C*2	6.6	22221	595071	3.73	4.8	554	311631	0.18
*P*1.000	3.4	118	616145	0.02	3.1	147	312947	0.05

**Table 2:** Diffraction data statistics for sets 1 and 2 in space group *I*4 Values in parentheses are for the last resolution shell.

	Data set 1	Data set 2
Resolution range (Å)	46.4–1.80 (1.86–1.80)	45.4–1.80 (1.864–1.80)
Space group	*I*4	*I*4
Unit-cell parameters (Å, °)	*a* = *b* = 95.60, *c* = 254.98, α = β = γ = 90	*a* = *b* = 96.55, *c* = 256.84, α = β = γ = 90
Total reflections	1462171 (72806)	787298 (50148)
Unique reflections	95273 (5642)	108128 (10687)
Multiplicity	15.3 (12.9)	7.3 (4.7)
Completeness (%)	90.70 (54.07)†	99.83 (98.36)
Mean *I*/σ(*I*)	34.72 (4.13)	18.44 (1.29)
Wilson *B* factor (Å^2^)	20.33	25.76
*R*_merge_	0.069 (0.6876)	0.081 (1.097)
CC_1/2_	1 (0.894)	0.999 (0.467)

† The data set is more than 98% complete at a resolution of 2.0 Å.

**Table 3:** Twinning analysis of both sets processed in *I*4

	Observed		Theoretical	
	Set 1 (*I*4)	Set 2 (*I*4)	Untwinned	Perfect twin
Wilson ratio				
〈*I* ^2^〉/〈*I*〉^2^	2.116	2.182	2.0	1.5
Padilla and Yeates statistics (Padilla & Yeates, 2003 ▶)				
〈|*L*|〉	0.497	0.504	0.5	0.375
〈*L* ^2^〉	0.329	0.337	0.333	0.2

**Table 4:** Occupancies of alternately positioned molecules

	Set 1			Set 2		
	*REFMAC*		*MAIN*	*REFMAC*		*MAIN*
Molecule	Occupancy	*B* factor (Å^2^)	Occupancy	Occupancy	*B* factor (Å^2^)	Occupancy
1–4, 6–9	—	25.9 ± 14.0	—	—	30.1 ± 15.0	—
0*A*	0.42	25.8 ± 9.9	0.40	0.47	30.6 ± 14.2	0.47
0*B*	0.58	26.0 ± 13.2	0.60	0.53	30.7 ± 13.6	0.53
5*A*	0.49	33.4 ± 12.1	0.49	0.51	41.9 ± 13.6	0.50
5*B*	0.51	33.2 ± 11.9	0.51	0.49	41.3 ± 13.0	0.50

**Table 5:** Refinement statistics

Resolution range (Å)	46.37–1.80 (1.86–1.80)
*R*_work_	0.190 (0.211)
*R*_free_	0.234 (0.269)
No. of non-H atoms	
Total	9787
Macromolecules	9004
Ligands	20
Water	763
No. of protein residues	910
R.m.s.d., bonds (Å)	0.016
R.m.s.d., angles (°)	1.71
Average *B* factor (Å^2^)	
Overall	24
Macromolecules	23.3
Ligands	48
Solvent	31

## References

[bb1] Adams, P. D. *et al.* (2010). *Acta Cryst.* D**66**, 213–221.

[bb2] Afonine, P. V., Grosse-Kunstleve, R. W., Chen, V. B., Headd, J. J., Moriarty, N. W., Richardson, J. S., Richardson, D. C., Urzhumtsev, A., Zwart, P. H. & Adams, P. D. (2010). *J. Appl. Cryst.* **43**, 669–676. 10.1107/S0021889810015608 PMC2906258 20648263

[bb3] Berman, H. M., Westbrook, J., Feng, Z., Gilliland, G., Bhat, T. N., Weissig, H., Shindyalov, I. N. & Bourne, P. E. (2000). *Nucleic Acids Res.* **28**, 235–242. 10.1093/nar/28.1.235 PMC102472 10592235

[bb4] Brünger, A. T., Adams, P. D., Clore, G. M., DeLano, W. L., Gros, P., Grosse-Kunstleve, R. W., Jiang, J.-S., Kuszewski, J., Nilges, M., Pannu, N. S., Read, R. J., Rice, L. M., Simonson, T. & Warren, G. L. (1998). *Acta Cryst.* D**54**, 905–921. 10.1107/s0907444998003254 9757107

[bb5] Dornberger-Schiff, K. (1956). *Acta Cryst.* **9**, 593–601.

[bb6] Dornberger-Schiff, K. & Grell-Niemann, H. (1961). *Acta Cryst.* **14**, 167–177.

[bb7] Emsley, P., Lohkamp, B., Scott, W. G. & Cowtan, K. (2010). *Acta Cryst.* D**66**, 486–501. 10.1107/S0907444910007493 PMC2852313 20383002

[bb8] Fisher, R. G. & Sweet, R. M. (1980). *Acta Cryst.* A**36**, 755–760.

[bb9] Giacovazzo, C. (2002). *Fundamentals of Crystallography* Chester, Oxford: IUCr/Oxford University Press.

[bb10] Hare, S., Cherepanov, P. & Wang, J. (2009). *Acta Cryst.* D**65**, 966–973. 10.1107/S0907444909023695 19690374

[bb11] Helliwell, J. R. (2008). *Crystallogr. Rev.* **14**, 189–250.

[bb12] Hwang, W. C., Lin, Y., Santelli, E., Sui, J., Jaroszewski, L., Stec, B., Farzan, M., Marasco, W. A. & Liddington, R. C. (2006). *J. Biol. Chem.* **281**, 34610–34616. 10.1074/jbc.M603275200 PMC7981188 16954221

[bb13] Janowski, R., Kozak, M., Jankowska, E., Grzonka, Z., Grubb, A., Abrahamson, M. & Jaskolski, M. (2001). *Nature Struct. Biol.* **8**, 316–320. 10.1038/86188 11276250

[bb14] Jenko Kokalj, S., Gunčar, G., Štern, I., Morgan, G., Rabzelj, S., Kenig, M., Staniforth, R. A., Waltho, J. P., Žerovnik, E. & Turk, D. (2007). *J. Mol. Biol.* **366**, 1569–1579. 10.1016/j.jmb.2006.12.025 17217964

[bb15] Kabsch, W. (2010). *Acta Cryst.* D**66**, 125–132. 10.1107/S0907444909047337 PMC2815665 20124692

[bb16] Kamtekar, S., Berman, A. J., Wang, J., Lázaro, J. M., de Vega, M., Blanco, L., Salas, M. & Steitz, T. A. (2004). *Mol. Cell*, **16**, 609–618. 10.1016/j.molcel.2004.10.019 15546620

[bb17] Krissinel, E. & Henrick, K. (2007). *J. Mol. Biol.* **372**, 774–797. 10.1016/j.jmb.2007.05.022 17681537

[bb18] Krug, M., Weiss, M. S., Heinemann, U. & Mueller, U. (2012). *J. Appl. Cryst.* **45**, 568–572.

[bb19] Lebedev, A. A., Vagin, A. A. & Murshudov, G. N. (2006). *Acta Cryst.* D**62**, 83–95. 10.1107/S0907444905036759 16369097

[bb20] McCoy, A. J., Grosse-Kunstleve, R. W., Adams, P. D., Winn, M. D., Storoni, L. C. & Read, R. J. (2007). *J. Appl. Cryst.* **40**, 658–674. 10.1107/S0021889807021206 PMC2483472 19461840

[bb21] Mueller, U., Darowski, N., Fuchs, M. R., Förster, R., Hellmig, M., Paithankar, K. S., Pühringer, S., Steffien, M., Zocher, G. & Weiss, M. S. (2012). *J. Synchrotron Rad.* **19**, 442–449. 10.1107/S0909049512006395 PMC3408958 22514183

[bb22] Murshudov, G. N., Skubák, P., Lebedev, A. A., Pannu, N. S., Steiner, R. A., Nicholls, R. A., Winn, M. D., Long, F. & Vagin, A. A. (2011). *Acta Cryst.* D**67**, 355–367. 10.1107/S0907444911001314 PMC3069751 21460454

[bb23] Otwinowski, Z. & Minor, W. (1997). *Methods Enzymol.* **276**, 307–326. 10.1016/S0076-6879(97)76066-X 27754618

[bb24] Padilla, J. E. & Yeates, T. O. (2003). *Acta Cryst.* D**59**, 1124–1130. 10.1107/s0907444903007947 12832754

[bb25] Pletnev, S., Morozova, K. S., Verkhusha, V. V. & Dauter, Z. (2009). *Acta Cryst.* D**65**, 906–912. 10.1107/S0907444909020927 PMC2733879 19690368

[bb26] Rabzelj, S., Turk, V. & Zerovnik, E. (2005). *Protein Sci.* **14**, 2713–2722. 10.1110/ps.051609705 PMC2253288 16155205

[bb27] Rabzelj, S., Viero, G., Gutiérrez-Aguirre, I., Turk, V., Dalla Serra, M., Anderluh, G. & Zerovnik, E. (2008). *FEBS J.* **275**, 2455–2466. 10.1111/j.1742-4658.2008.06390.x 18397316

[bb28] Robbins, A. H., Domsic, J. F., Agbandje-McKenna, M. & McKenna, R. (2010*a*). *Acta Cryst.* D**66**, 628–634. 10.1107/S0907444910006797 PMC2865368 20445238

[bb29] Robbins, A. H., Domsic, J. F., Agbandje-McKenna, M. & McKenna, R. (2010*b*). *Acta Cryst.* D**66**, 950–952. 10.1107/S0907444910023723 PMC2917278 20693695

[bb30] Rye, C. A., Isupov, M. N., Lebedev, A. A. & Littlechild, J. A. (2007). *Acta Cryst.* D**63**, 926–930. 10.1107/S0907444907026315 17642519

[bb31] Sheldrick, G. M. (2008). *Acta Cryst.* A**64**, 112–122. 10.1107/S0108767307043930 18156677

[bb32] Stevens, R. C., Yokoyama, S. & Wilson, I. A. (2001). *Science*, **294**, 89–92. 10.1126/science.1066011 11588249

[bb33] Stubbs, M. T., Laber, B., Bode, W., Huber, R., Jerala, R., Lenarcic, B. & Turk, V. (1990). *EMBO J.* **9**, 1939–1947. 10.1002/j.1460-2075.1990.tb08321.x PMC551902 2347312

[bb34] Tanaka, S., Kerfeld, C. A., Sawaya, M. R., Cai, F., Heinhorst, S., Cannon, G. C. & Yeates, T. O. (2008). *Science*, **319**, 1083–1086. 10.1126/science.1151458 18292340

[bb35] Terwilliger, T. C. (2011). *J. Struct. Funct. Genomics*, **12**, 43–44. 10.1007/s10969-011-9114-2 PMC3123458 21681528

[bb36] Trame, C. B. & McKay, D. B. (2001). *Acta Cryst.* D**57**, 1079–1090. 10.1107/s0907444901007673 11468391

[bb37] Tsai, Y., Sawaya, M. R. & Yeates, T. O. (2009). *Acta Cryst.* D**65**, 980–988. 10.1107/S0907444909025153 19690376

[bb38] Turk, D. (2013). *Acta Cryst.* D**69**, 1342–1357. 10.1107/S0907444913008408 PMC3727325 23897458

[bb39] Wang, J., Kamtekar, S., Berman, A. J. & Steitz, T. A. (2005). *Acta Cryst.* D**61**, 67–74. 10.1107/S0907444904026721 15608377

[bb40] Wang, J., Rho, S.-H., Park, H. H. & Eom, S. H. (2005). *Acta Cryst.* D**61**, 932–941. 10.1107/S0907444905009546 15983416

[bb41] Winn, M. D. *et al.* (2011). *Acta Cryst.* D**67**, 235–242.

[bb42] Yeates, T. O. (1997). *Methods Enzymol.* **276**, 344–358. 9048378

[bb43] Zerovnik, E., Pompe-Novak, M., Skarabot, M., Ravnikar, M., Musevic, I. & Turk, V. (2002). *Biochim. Biophys. Acta*, **1594**, 1–5. 10.1016/s0167-4838(01)00295-3 11825603

[bb44] Zhu, X., Xu, X. & Wilson, I. A. (2008). *Acta Cryst.* D**64**, 843–850. 10.1107/S0907444908016648 PMC2631119 18645233

