# Supplementary material for: Partial rotational lattice order–disorder in stefin B crystals
Source: Acta Crystallogr D Biol Crystallogr. 2014 Mar 19;70(Pt 4):1015–25. doi: 10.1107/S1399004714000091 (PMC3975888; doi:10.1107/S1399004714000091)
Supplement: Supplementary file 1 [file d-70-01015-sup1.pdf]

## Supporting Information

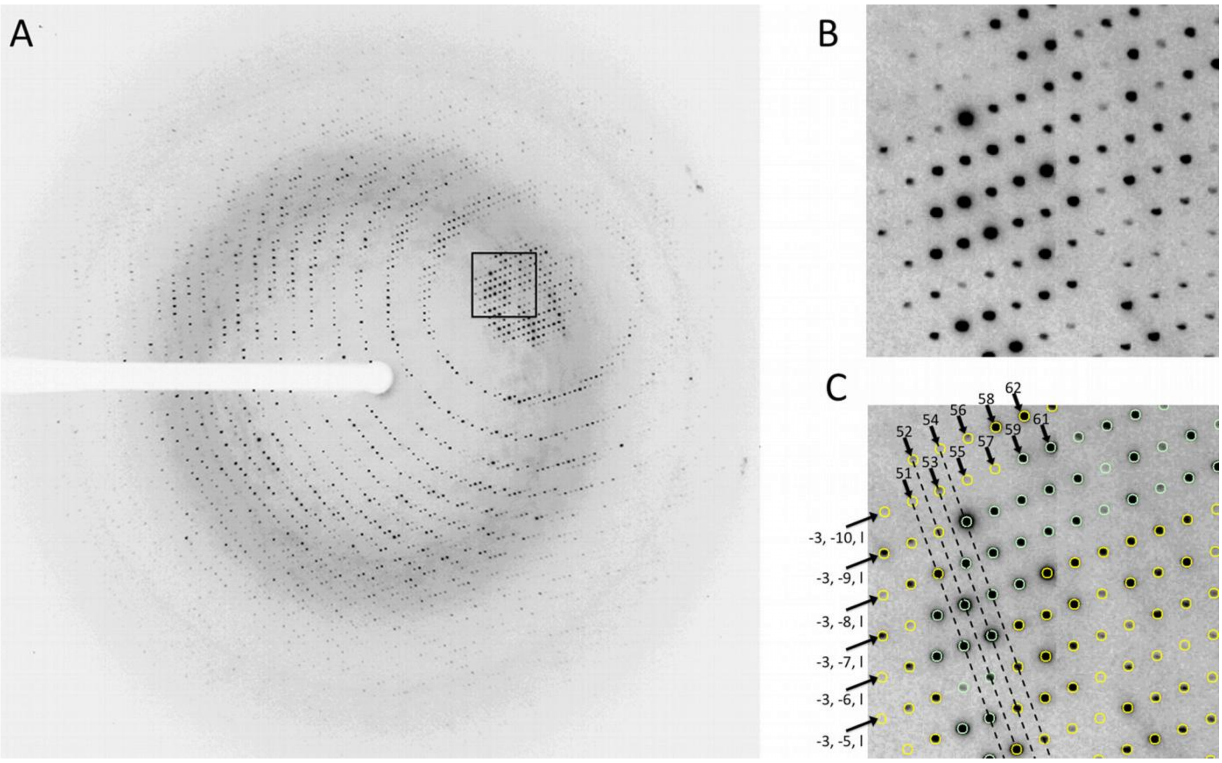


**Supporting Figure S1**. The diffraction pattern of stefin B crystal 2. A) Complete diffraction image, B) enlarged section and C) the same enlarged section after integration with h, k, and l indices indicating the positions of predicted reflections. Figure was prepared with HKL 2000 (Otwinowski & Minor, 1997).

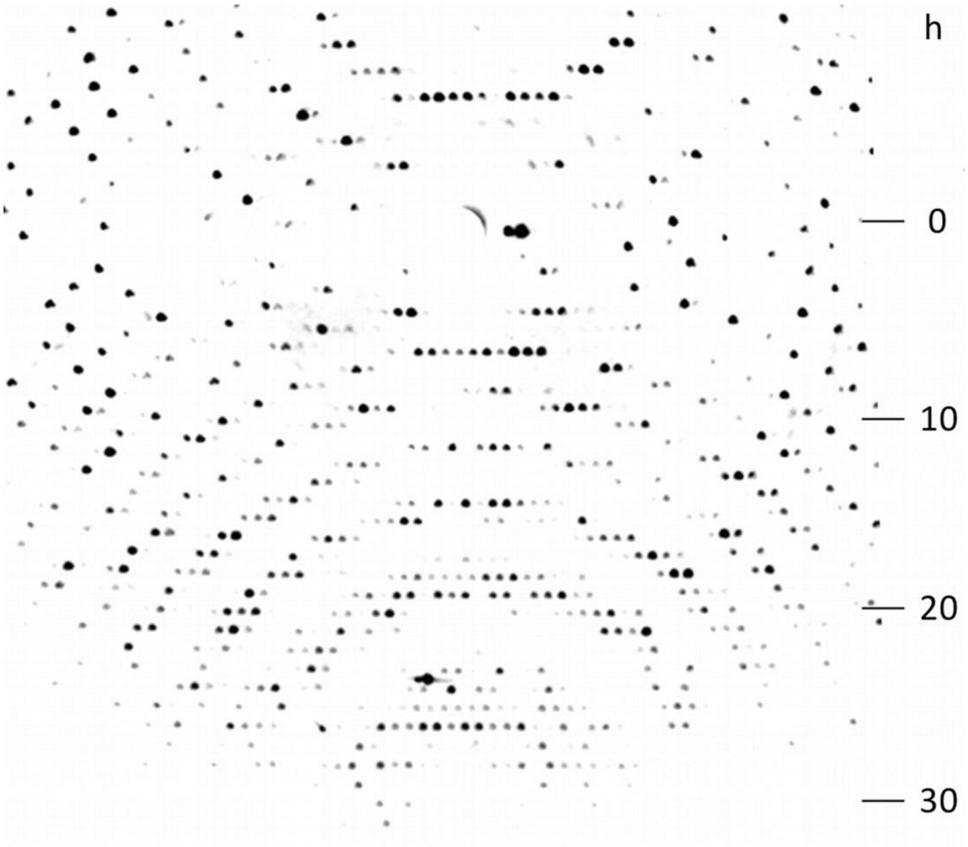


**Supporting Figure S2**. The diffraction pattern of an additional crystal of stefin B. Diffraction image was taken in the orientation in which the c* axis coincided with the rotation axis. The image was collected using Proteum X8 (Bruker AXS) using 2theta offset of 10 degrees and 5 minute exposure time, with crystal-to-detector distance of 110 mm. The resolution limit corresponds to 2.8Å.

| Space group | P 1 | C 1 2 1 | F 2 2 2 | I 4 | I 4 2 2 |
|---|---|---|---|---|---|
| Resolution (Å) | 20.0 - 2.50 (2.59 - 2.50) | 20.0 - 2.50 (2.59 - 2.50) | 20.0 - 2.50 (2.59 - 2.50) | 20.0 - 2.50 (2.59 - 2.50) | 20.0 - 2.50 (2.59 - 2.50) |
| Unit cell | | | | | |
| a, b, c (Å) | 95.65, 95.52, 144.25 | 272.21, 95.65, 95.52 | 135.20, 135.19, 255.02 | 95.60, 95.60, 255.03 | 95.60, 95.60, 255.03 |
| a, b, g (˚) | 109.26, 109.34, 90.01 | 90, 110.47, 90 | 90, 90, 90 | 90, 90, 90 | 90, 90, 90 |
| | | | | | |
| Total reflections | 616144 (60020) | 595070 (60199) | 590759 (59229) | 621836 (61457) | 598276 (61250) |
| Unique reflections | 152656 (14837) | 78377 (7630) | 39725 (3804) | 39191 (3869) | 20833 (2017) |
| Multiplicity | 4.0 (4.0) | 7.6 (7.9) | 14.9 (15.6) | 15.9 (15.9) | 28.7 (30.4) |
| Completeness (%) | 98.34 (95.83) | 98.96 (97.07) | 99.02 (96.35) | 99.32 (98.35) | 99.05 (98.06) |
| Mean I/sigma(I) | 26.49 (13.36) | 35.88 (18.57) | 50.09 (25.96) | 52.06 (26.06) | 68.81 (35.76) |
| Wilson B-factor | 22.57 | 22.76 | 23.25 | 22.68 | 23.79 |
| R-merge | 0.03374 (0.08498) | 0.06582 (0.127) | 0.06968 (0.1374) | 0.04652 (0.1084) | 0.07183 (0.1402) |
| R-meas | 0.03891 | 0.07055 | 0.07212 | 0.04805 | 0.07309 |
| CC1/2 | 0.999 (0.993) | 0.998 (0.992) | 0.999 (0.995) | 1 (0.997) | 1 (0.998) |
| CC* | 1 (0.998) | 1 (0.998) | 1 (0.999) | 1 (0.999) | 1 (0.999) |

**Supporting Table S1**. The diffraction data and scaling statistics tables for data from crystals 1 truncated at 2.5 Å.

Data were processed in P1, C2, F222, I4, and I422 space groups. To compensate for the differences between the two crystals in the data completeness at higher resolution shells only the low resolution data (20 – 2.5 Å) were used. Default error scale factor of 1.3 and error zone of 0.03 were used in scaling with the HKL2000 program (Otwinowski & Minor, 1997). The unmerged intensities from HKL2000 were used for calculation of statistical parameters with Phenix (Adams et al., 2010).

| Space group | P 1 | C 1 2 1 | F 2 2 2 | I 4 | I 4 2 2 |
|---|---|---|---|---|---|
| Resolution (Å) | 20.0 - 2.50 (2.59 - 2.50) | 20.0 - 2.50 (2.59 - 2.50) | 20.0 - 2.50 (2.59 - 2.50) | 20.0 - 2.50 (2.59 - 2.50) | 20.0 - 2.50 (2.59 - 2.50) |
| Unit cell | | | | | |
| a, b, c (Å) | 96.59, 96.52, 145.37 | 136.65, 136.44, 145.35 | 136.50, 136.68, 256.85 | 96.55, 96.55, 256.84 | 96.55, 96.55, 256.84 |
| a, b, g (˚) | 109.38, 109.40, 89.92 | 90, 117.97, 90 | 90, 90, 90 | 90, 90, 90 | 90, 90, 90 |
| | | | | | |
| Total reflections | 312946 (30964) | 311630 (30801) | 310501 (30825) | 309678 (30992) | 309280 (30990) |
| Unique reflections | 157382 (15550) | 80230 (7896) | 41273 (4053) | 40168 (3997) | 21396 (2089) |
| Multiplicity | 2.0 (2.0) | 3.9 (3.9) | 7.5 (7.6) | 7.7 (7.8) | 14.5 (14.8) |
| Completeness (%) | 98.41 (96.79) | 99.28 (97.75) | 99.82 (98.49) | 99.91 (99.28) | 99.89 (99.24) |
| Mean I/sigma(I) | 16.40 (7.09) | 22.98 (9.97) | 31.86 (13.94) | 32.43 (14.17) | 44.23 (19.56) |
| Wilson B-factor | 28.88 | 29.05 | 29.59 | 29.27 | 30.39 |
| R-merge | 0.03083 (0.09338) | 0.04788 (0.1197) | 0.0609 (0.134) | 0.06179 (0.1352) | 0.06644 (0.1418) |
| R-meas | 0.0436 | 0.05557 | 0.06547 | 0.0663 | 0.06892 |
| CC1/2 | 0.998 (0.971) | 0.998 (0.984) | 0.998 (0.991) | 0.999 (0.991) | 0.999 (0.995) |
| CC* | 0.999 (0.993) | 1 (0.996) | 1 (0.998) | 1 (0.998) | 1 (0.999) |

**Supporting Table S2**. The diffraction data and scaling statistics tables for data from crystals 2 truncated at 2.5 Å in  P1, C2, F222, I4, and I422.

To compensate for the differences between the two crystals in the data completeness at higher resolution shells, only the low resolution data (20 – 2.5 Å) were used. Default error scale factor of 1.3 and error zone of 0.03 were used in scaling with the HKL2000 suite. Unmerged intensities from HKL2000 (Otwinowski & Minor, 1997) were used for statistic calculation with Phenix (Adams et al., 2010).

## Supporting References


Adams, P. D., Afonine, P. V., Bunkoczi, G., Chen, V. B., Davis, I. W., Echols, N., Headd, J. J., Hung, L. W., Kapral, G. J., Grosse-Kunstleve, R. W., McCoy, A. J., Moriarty, N. W., Oeffner, R., Read, R. J., Richardson, D. C., Richardson, J. S., Terwilliger, T. C. & Zwart, P. H. (2010). *Acta Cryst.* D**66**, 213-221.

Otwinowski, Z. & Minor, W. (1997). *Methods Enzymol* **276**, 307-326.
